# Epigene functional diversity: isoform usage, disordered domain content, and variable binding partners

**DOI:** 10.1186/s13072-025-00571-z

**Published:** 2025-02-01

**Authors:** Leroy Bondhus, Aileen A. Nava, Isabelle S. Liu, Valerie A. Arboleda

**Affiliations:** 1 Department of Human Genetics, David Geffen School of Medicine, UCLA, 615 Charles E. Young Drive South, Los Angeles, CA 90095 USA; 2 Department of Pathology and Laboratory Medicine, David Geffen School of Medicine, UCLA, Los Angeles, CA 90095 USA; 3 Department of Computational Medicine, David Geffen School of Medicine, UCLA, Los Angeles, CA 90095 USA; 4 Molecular Biology Institute, UCLA, Los Angeles, CA 90095 USA; 5 Jonsson Comprehensive Cancer Center, UCLA, Los Angeles, CA 90095 USA

**Keywords:** Transcriptomics, Epigenes, Chromatin modifiers, Epigenetics, Rare diseases

## Abstract

**Background:**

Epigenes are defined as proteins that perform post-translational modification of histones or DNA, reading of post-translational modifications, form complexes with epigenetic factors or changing the general structure of chromatin. This specialized group of proteins is responsible for controlling the organization of genomic DNA in a cell-type specific fashion, controlling normal development in a spatial and temporal fashion. Moreover, mutations in epigenes have been implicated as causal in germline pediatric disorders and as driver mutations in cancer. Despite their importance to human disease, to date, there has not been a systematic analysis of the sources of functional diversity for epigenes at large. Epigenes’ unique functions that require the assembly of pools within the nucleus suggest that their structure and amino acid composition would have been enriched for features that enable efficient assembly of chromatin and DNA for transcription, splicing, and post-translational modifications.

**Results:**

In this study, we assess the functional diversity stemming from gene structure, isoforms, protein domains, and multiprotein complex formation that drive the functions of established epigenes. We found that there are specific structural features that enable epigenes to perform their variable roles depending on the cellular and environmental context. First, epigenes are significantly larger and have more exons compared with non-epigenes which contributes to increased isoform diversity. Second epigenes participate in more multimeric complexes than non-epigenes. Thirdly, given their proposed importance in membraneless organelles, we show epigenes are enriched for substantially larger intrinsically disordered regions (IDRs). Additionally, we assessed the specificity of their expression profiles and showed epigenes are more ubiquitously expressed consistent with their enrichment in pediatric syndromes with intellectual disability, multiorgan dysfunction, and developmental delay. Finally, in the L1000 dataset, we identify drugs that can potentially be used to modulate expression of these genes.

**Conclusions:**

Here we identify significant differences in isoform usage, disordered domain content, and variable binding partners between human epigenes and non-epigenes using various functional genomics datasets from Ensembl, ENCODE, GTEx, HPO, LINCS L1000, and BrainSpan. Our results contribute new knowledge to the growing field focused on developing targeted therapies for diseases caused by epigene mutations, such as chromatinopathies and cancers.

**Supplementary Information:**

The online version contains supplementary material available at 10.1186/s13072-025-00571-z.

## Introduction

The information encoded in the genome must be tightly regulated throughout organism development to give rise to the diversity of cell types and higher order biological structures that characterize complex multicellular life. As the sequence of the genome is essentially invariant over the life of the organism, this regulation is achieved in large part by modification to the secondary chemical and physical structure of the genome: the epigenome. Epigenes then are those genes involved in the regulation, modification, and maintenance of the epigenome, the diverse functions of which we previously reviewed in Nava et al. [1].

Beyond epigenes' importance in the fundamental biological process of genome regulation, epigenes also have particular relevance to human health. Somatic mutations in epigenes contribute to the development of numerous cancers and are often important prognostic markers for disease [2–7]. Germline mutations in a subset of epigenes are also associated with a diverse set of complex syndromic disorders [1, 8, 9]. In contrast to other genetic disorders which directly affect one or a few body systems, many epigene-related diseases affect numerous distinct body systems. For instance, Arboleda-Tham syndrome is characterized by a combination of neurodevelopmental, cardiac, and gastrointestinal abnormalities, along with global effects such as general hypotonia [10, 11]. Whether this tendency to affect multiple body systems is related to epigenes having more widespread expression or a result of their functioning in very early developmental processes in progenitor cells remains to be fully elucidated. It is also worth noting that not all genes essential for organismal development will be associated with disease-causing mutations. Mutations in genes essential for early development may be embryonic lethal [12].

The human genome encodes relatively few epigenes compared to, for instance, transcription factors [13]. The epigenetic code that epigenes must establish and maintain is complex and involves a variety of distinct histone marks [14, 15], precise positioning of nucleosomes [16, 17], and higher levels of organization such as those brought about by enhancer-promoter interactions [18] or topologically associating domains [19, 20]. The epigenetic code is responsible for establishing and maintaining the many distinct cell identities that exist throughout the organism and its development [21].

Epigenes in general may target a range of genomic regions, produce distinct epigenetic modifications, or respond to different signals within the cell. A variety of mechanisms are known which can expand the functional range of individual genes and which, in the case of epigenes, may help explain how they are able to produce the complex epigenetic structure of the cell. Individual genes often encode a variety of isoforms with distinct functional potentials [22]. For instance the histone deacetylase (HDAC) cofactor *SIN3A* is known to be expressed as alternative isoforms with and without a HDAC binding domain modifying its chromatin repressor activity [23], and isoforms of *DPF2*, a subunit of the BAF chromatin remodeling complex, have been shown to have modulate the genomic binding pattern of its protein complex [24]. In addition to encoding multiple distinct isoforms, the protein a gene encodes may bind to a variety of different partners that modulate their activity [25, 26]. Indeed, many epigenes are known to function in the context of multiprotein complexes, with different complex members contributing different functional modalities. For instance, the KAT6A protein is known to participate in at least three distinct complexes, distinguished by the species of BRPF protein they incorporate each of which contributes to the complex DNA and histone binding modalities; little is known however about the differences in function for these complexes [27]. The contribution of these various sources of functional complexity has not been systematically investigated for epigenes.

In this study, we aim to identify sources of functional diversity for epigenes and highlight how such diversity might contribute to the clinical phenotypes observed in epigene mutations. In particular, we look at the potential contribution of isoform usage and the protein compositional diversity of epigene-related multiprotein complexes. Given the emerging role of disordered domains in guiding a variety of functions for nuclear organization and gene regulation, for instance by mediating protein–RNA interactions, we investigate the prevalence and characteristics of disordered domains in epigenes. Finally, we look at the expression of epigenes together with the phenotypic complexity of their associated disorders and mention some of the tools available to modulate their expression that may aid in deeper investigations of their functions or in the development of treatments for their associated disorders.

## Results

### Epigene curation

As has been previously defined, ‘epigenes’ are a broad class of genes which are recruited to specific genomic regions to facilitate modification and maintenance of epigenetic state [1, 28]. These epigenetic-related events often require the binding of multiple distinct proteins into larger complexes that often modulate: where in the genome the epigenes' effect is targeted [29], what modification will be added [30–32], and what signals the epigene will respond to [33–35]. In our previous work [1], we curated epigenes based on the ability of protein-coding human genes to influence the epigenome of a cell, while excluding histone-coding and protamine-coding genes [28], resulting in a list of 720 epigenes. While histone-coding genes are foundational to the epigenome and its regulation, and variant histone proteins are known to modulate various epigenetic processes [36], we argue for the conceptual distinction of epigenes as those involved in the regulation of epigenetic architecture apart from those which are the substrate of epigenetic architectures such as histones. Our separate treatment of the histones from epigenes also had a substantial technical basis, which is that accounting for histones in genetic analysis is notoriously complex as they have an extreme degree of gene redundancy, with multiple copies of genes existing encoding identical protein products [37, 38]. The genes we included in our epigene set encompass the more stringently defined chromatinopathy gene sets that make up the mendelian disorders of the epigenetic machinery [9], which are limited to proteins that function as readers, writers, erasers, remodelers and insulators or any combination of these. As histones are foundational for epigenomic structure, for various analyses we included these as an additional contrast set with the epigene set.

### Epigenes are significantly longer genes, transcripts, and have a greater number of exons

**Fig. 1:**
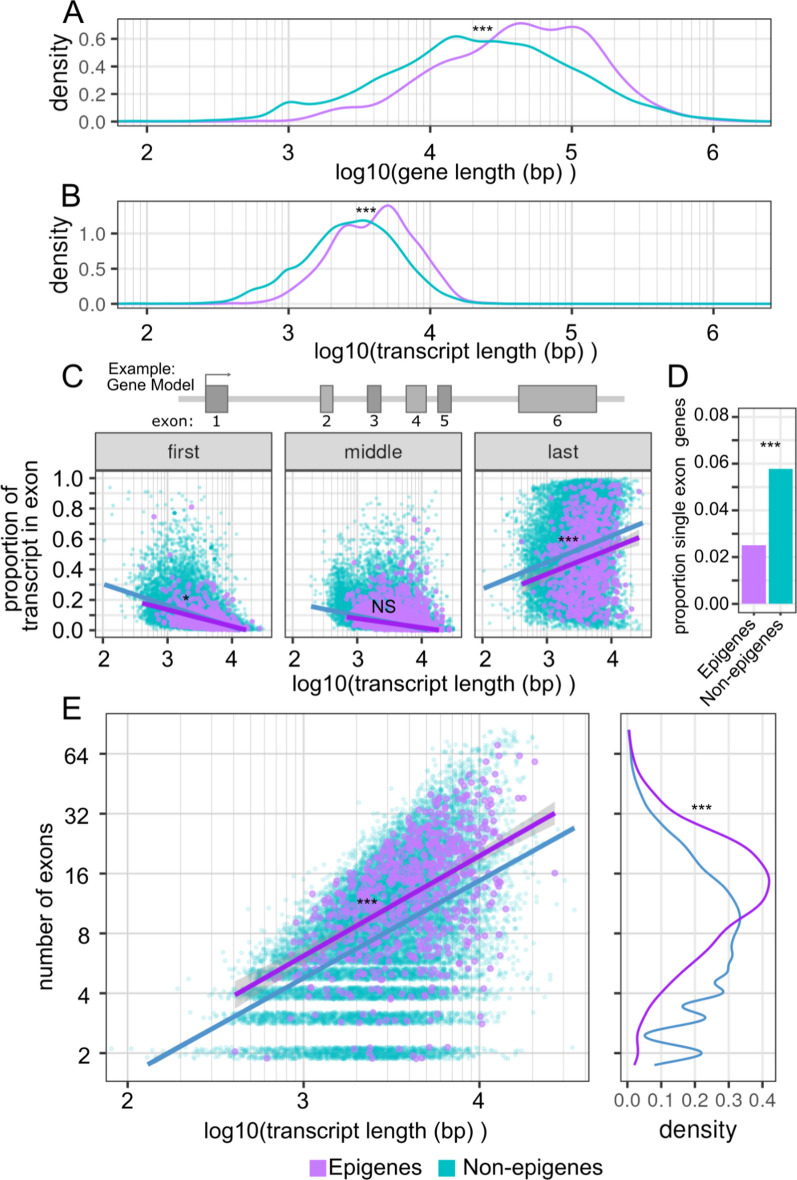
**Epigenes are larger and have more exons than other genes**. **A** Density distribution of gene lengths for epigenes and all other genes. **B** Density distribution of transcript lengths for epigenes and all other genes. **C** Transcript length plotted against the proportion of the transcript in each exon partitioning by first, middle, and last exons. Each point represents a single exon from a canonical transcript. Regression lines shown for epigene and all other genes groups. Genes encoded by a single exon were excluded. **D** Proportion of epigenes and other genes that are encoded by single exon genes. **E** Transcript length against exon count for each gene. Only the canonical transcript is considered. Regression lines shown for epigene and all other gene groups. Single exon genes were excluded. Significance level is indicated by asterisks: NS = not significant, * < 0.05, ** < 0.01, *** < 0.001

We began our characterization of epigenes by looking at the gene structure of epigenes. Structural annotations for genes were obtained from Ensembl [39, 40]. We observed that the gene size for epigenes was substantially longer compared with all non-epigenes (epigene: geometric mean = 41.4 kb, interquartile range (IQR) = 17.4 to 107.5 kb; non-epigene: geometric mean = 21.8 kb, IQR = 8.3 kb to 62.7 kb; *p* < 1e−5, permutation test) (Fig. 1A). Similarly, epigenes mRNA transcripts were also significantly longer than those of non-epigenes (epigene: geometric mean = 4.0 kb, IQR = 2.5 to 6.3 kb; non-epigene: geometric mean = 2.7 kb, IQR = 1.7 to 4.7 kb; *p* < 1e−5, permutation test) (Fig. 1B). This is in contrast to canonical and variant histones, which are small genes (geometric mean: 1.5 kb, IQR = 495 bp to 2.9 kb) that encode correspondingly small transcripts (geometric mean: 603 bp, IQR = 469 to 633 bp) (Figure S1A, B). As a note, we use the geometric mean for comparison where distributions are approximately log normal; the geometric mean being a measure of central tendency on the log scale [41].

The increase in transcript length for epigenes was not attributable to an increase in exon size, and in fact we observed that in epigenes first and last exons tended to account for an overall smaller proportion of the transcript length than those same exons in non-epigenes (*p* = 2.7e−5, *p* = 0.11, *p* < 1e−5, permutation test. First, middle, last exon respectively) (Fig. 1C, Table S1). Single exon genes make up a disproportionately small minority of epigenes (2.5%, 18/720) compared to all non-epigenes (5.8%, 1117/19,329) (*p* < 2.2e−16, two-sample test of proportions) (Fig. 1D). This is in contrast to histones, the vast majority (86%, 86/100) of which are single exon genes (Figure S1C). We excluded single exon genes and histones from the analysis of exon structure. When we regressed the proportion of the transcript encoded by exons against overall transcript size, partitioned for "first',"middle", and "last" exons, we observed that the last exon had a strong positive correlation to overall transcript size (similar for both epigenes and non-epigenes; epigenes R^2^ = 0.69; non-epigenes R^2^ = 0.71, *p* = 0.36 permutation test), as has been previously reported [42].

As the exons of epigenes were not longer in size than for non-epigenes, we asked whether epigenes have an inflated number of exons for a given transcript size. Indeed when we regressed the number of exons against the overall transcript length we found this to be the case with epigenes having an increased number of exons relative to non-epigenes for a given transcript length (Fig. 1E).

### Higher complexity of isoform expression for epigenes

**Fig. 2:**
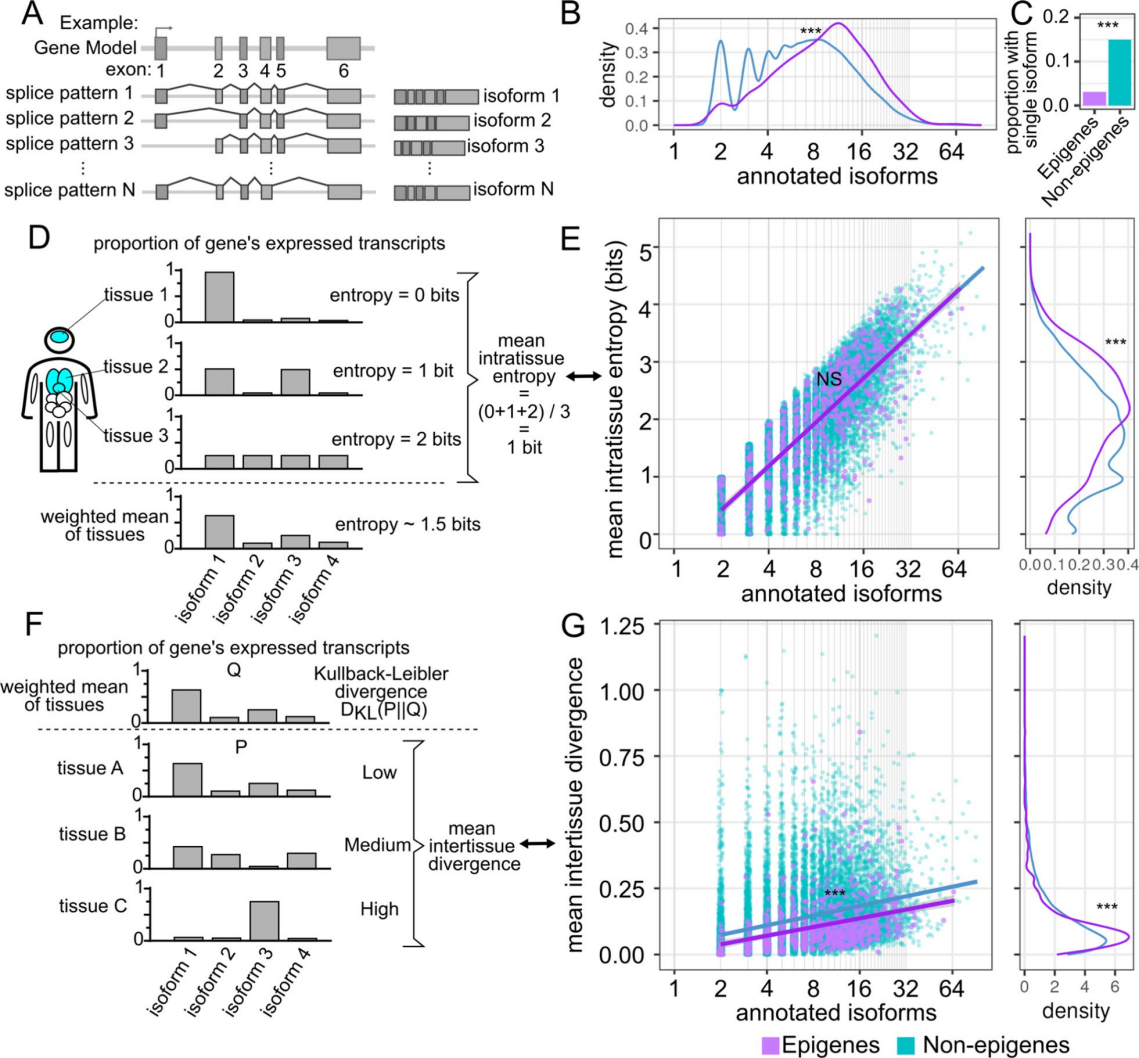
Epigenes have a greater number of expressed isoforms than other genes but a lower level of tissue specific patterns of relative isoform usage. (**A**) Toy diagram showing conceptual relation between exons, splice patterns, and isoforms for a single gene. (**B**) Density plot of the number of annotated isoforms associated with each gene. Single isoform genes were excluded. (**C**) Proportion of genes for which only a single isoform has been annotated. (**D**) Toy representation of entropy calculation. For each gene, the isoform proportion estimates are treated as a probability distribution on which entropy is calculated. Given a number of distinct isoforms, entropy is minimized when a single isoform dominates and is maximized as isoform expression proportions become uniform. See methods for precise method of calculation. (**E**) Number of annotated isoforms against mean intratissue entropy. Regression lines shown for epigene and all other genes groups. Density distribution of the entropy measure is shown to the right of the scatterplot. (**F**) Toy representation of Kullback-Leibler divergence, DKL(P||Q). For probability distribution P, DKL(P||Q) is minimized when P is equal to distribution Q, and increases as P becomes more dissimilar from Q. Here we define Q for a given gene as the weighted mean of all tissue or biosample isoform proportions. See methods for precise method of calculation. (**G**) Number of annotated isoforms against mean intertissue divergence as measured by Kullback-Leibler divergence. Regression lines shown for epigene and all other genes groups. Density distribution of the divergence measure is shown to the right of the scatterplot. Significance level is indicated by asterisks: *NS* not significant, *< 0.05, **< 0.01, ***< 0.001

We next investigated the potential functional consequence of this structural difference between epigenes and non-epigenes. Since epigenes have more exons for a given transcript length, we hypothesized that the increased exon count allows for larger possible combinations of exons and potentially increased transcriptional diversity of epigenes. The relation between exon structure, alternative splicing, and isoforms is illustrated in Fig. 2A.

In the Ensembl data, we could see that epigenes have an increased number of annotated isoforms (epigene: geometric mean = 8.8 isoforms, non-epigene: geometric mean = 6.5 isoforms, *p* < 1e−5 permutation test) (Fig. 2B) and a significantly smaller proportion of epigenes (3.1%, 22 of 720) were annotated as single-isoform genes compared to non-epigenes (17.7%, 2904 of 19,329) (*p* < 2.2e−16, 2-sample proportion test) (Fig. 2C). We wanted to further test whether there was evidence that these multiple transcripts contributed to the functional diversity of epigenes. For our isoform analyses, we excluded genes that were annotated as single isoform genes.

We next wanted to determine whether epigenes were expressing a greater diversity of isoforms compared with non-epigenes within a given tissue. For this analysis, we used transcript abundance estimates from the GTEx project [43, 44] which uses the RSEM algorithm [45] for transcript isoform abundance predictions from short-read RNA-seq data. We considered using long-read datasets to direct quantification of transcripts, but at this point, publicly available long-read data for large transcripts like epigenes are under-sequenced [46, 47]. We used two measures of functional diversity for isoform usage: (1) a measure of isoform diversity expressed within a biological context, e.g. intratissue or intracellular and (2) a measure of isoform diversity between distinct biological contexts. First, to measure functional diversity within tissue diversity, we used a weighted mean of intratissue entropies. Intratissue entropy balances, within a given tissue or biological sample, the number of expressed isoforms with their relative level of expression [48]. A basic intuition for the meaning of entropy here can be established by considering two behaviors of entropy. First, the minimum entropy occurs when only one isoform of a gene is expressed (Fig. 2D, entropy = 0 bits) while the maximum entropy for a given number of distinct isoforms occurs when all isoforms are equally expressed. This can be expressed in terms of bits, where bits are a unit of information in base 2, a syntax commonly used in computer science. For example, for equal expression of 4 distinct isoforms, entropy = 4 * −(¼ log_2_(¼)) = 2 bits, where bits are a unit of information in base 2. Entropy as measured in bits can also be considered as the average number of binary characters needed to represent a message made up of the results from a random draw of a gene's isoforms following their relative expression densities if we know a priori the number of draws that were made (e.g. if only one transcript, we need no additional information beyond that a transcript was drawn to know which transcript was pulled). Second, given the case where all isoforms of a gene are equally expressed, entropy will be greater for a gene with more isoforms than for a gene with fewer (see “Methods” section, Table S2).

For both epigenes and non-epigenes we found a strong correlation between the log number of annotated isoforms and mean intratissue entropy (R^2^ = 0.81 and R^2^ = 0.84 respectively), but no significant differences were identified between these two groups. Epigenes had a higher overall level of mean intratissue entropy compared to non-epigenes (epigenes: mean = 2.1 bits, sd = 0.92 bits; non-epigenes: mean = 1.7 bits, standard deviation (sd) = 0.94; *p* < 1e−5 permutation test) (Fig. 2E). For a gene with equal expression of all isoforms, these entropy values would correspond to 2^2.1^ = 4.3 unique isoforms and 2^1.7^ = 3.3 unique isoforms for epigenes and non-epigenes respectively. Thus within a given biological context, such as a tissue or cell, individual epigenes on average tend to express a greater diversity of isoforms than non-epigenes. Next we tested whether epigenes have a greater diversity of isoform expression profiles between different biological contexts than non-epigenes. For this, we used another measure based on entropy, Kulback-Leibler divergence (D_KL_) [49]. D_KL_ measures the dissimilarity between two probability distributions; by using this divergence metric we can compare for a given gene the isoform proportions expressed in one tissue with the typical proportions expressed across all tissues. If all tissues have similar isoform profiles, the mean D_KL_ will be relatively low, while if tissues have highly divergent profiles, the mean D_KL_ will be relatively high (Fig. 2F, “Methods” section).

Given our previous observation that epigenes had an overall higher level of intratissue entropy compared to non-epigenes, we were somewhat surprised to find that epigenes had a slightly depressed mean intertissue divergence relative to non-epigenes (epigenes: mean D_KL_ = 0.10, IQR = 0.05 to 0.13; non-epigenes: mean D_KL_ = 0.13, IQR = 0.05 to 0.18; *p* < 1e−5 permutation test) (Fig. 2G). Together these observations indicate that within tissues epigenes tend to express a greater diversity of isoforms, but between different tissues, epigenes tend to express isoform profiles that are more similar than non-epigenes.

We looked at relations of intratissue entropy and intertissue divergence with the specificity of gene expression and the number of paralogs each gene has in the human genome (Figure S2). Regressing the entropy measures against expression specificity, epigene status, and the interaction term, we observed that the specificity of gene expression had a modest and significant negative association with intratissue entropy (R^2^ = 0.09) and a small but significant positive association with intertissue divergence (R^2^ = 0.02). This indicates that genes with more specific patterns of expression tend to have lower average within tissue isoform diversity while also having a higher average between tissue divergence. However the binary epigene label (i.e. *is epigene* and *is not epigene*) did not have a significant effect for either of these regression models. In similar regressions of these entropy measures against the number of paralogs we also observed small but significant negative and positive associations with intratissue entropy (R^2^ = 0.007) and intertissue divergence (R^2^ = 0.006) respectively, again with no significant effect associated with the epigene label or the interaction term.

### Epigenes are associated with a large number of variable binding partners

**Fig. 3:**
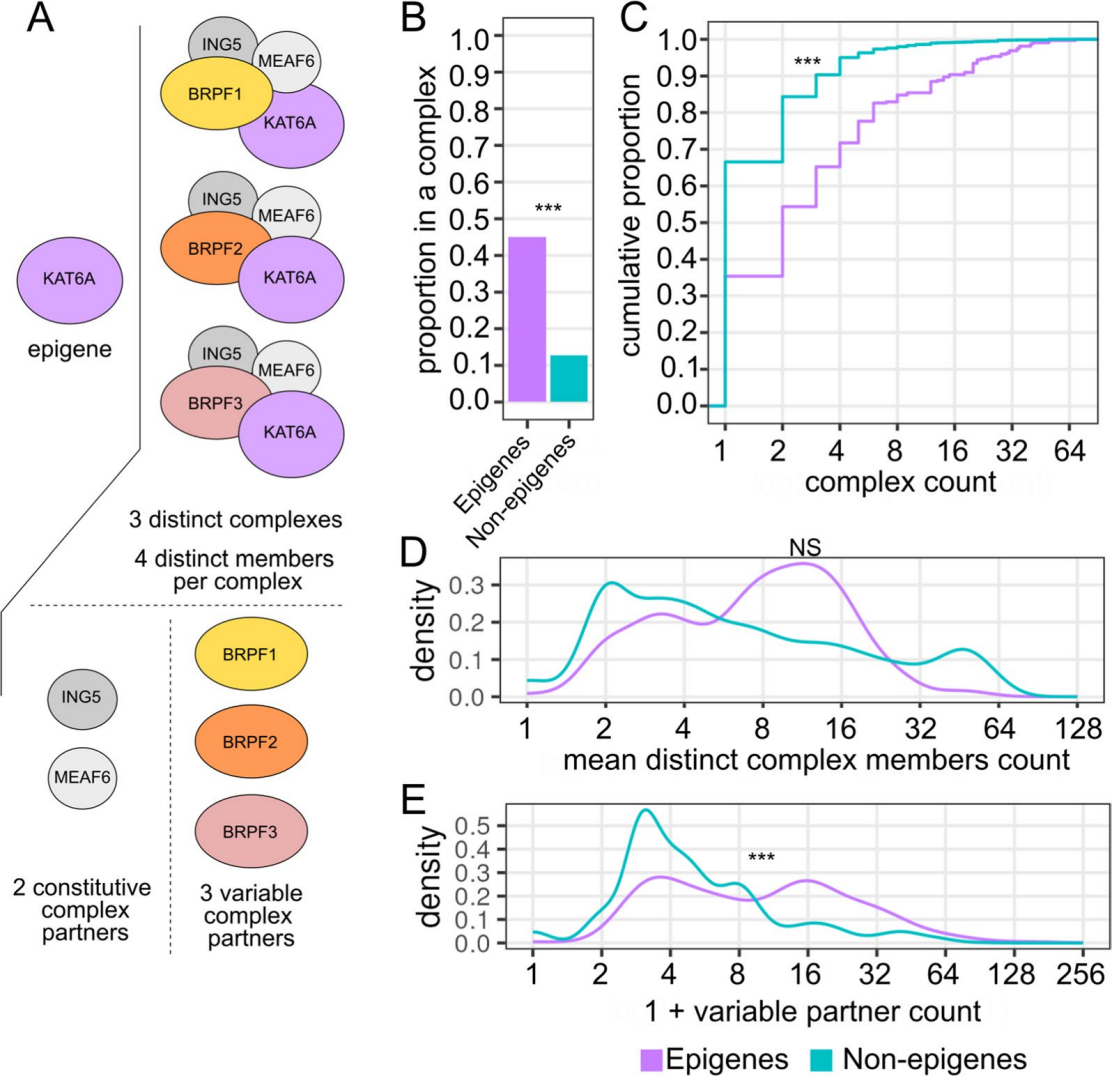
Epigenes have an increased number of variable binding partners compared with non-epigenes.** A** Toy figure demonstrating how distinct complexes and variable partners are counted. **B** Proportion of genes associated with some complex. **C** For genes associated with a protein complex, empirical cumulative density function (eCDF) of the number of variable complex partners for epigenes (purple) and all other genes (teal green). Excluded are all genes not associated with any complex. **D** Density of mean number of distinct proteins in complexes associated with each gene. For example, in the KAT6A example, there are 3 complexes associated with KAT6A, each of which has 4 distinct proteins, so the mean would be 4 proteins per complex for KAT6A. **E** Density of number of variable partners associated with each gene, excluding genes associated with one or fewer complexes. Significance level is indicated by asterisks: NS = not significant, * < 0.05, ** < 0.01, *** < 0.001

In addition to isoform diversity, interactions with other proteins add another potential layer of complexity to the function of epigenes. In multiprotein complexes, a protein may have constitutive interaction partners, without which they cannot carry out their core functions, as well as variable partners which can modulate the function of the overall complex. This situation is exemplified in Fig. 3A for the KAT6A (a.k.a. MOZ, MYST3) where KAT6A participates in three distinct complexes, each with ING5 and MEAF6, distinguished by the species of BRPF they incorporate, one of either BRPF1, BRPF2, or BRPF3 [27].

To investigate epigene complexes, we pulled annotated protein complex data from the EMBL-EBI Complex Portal which includes a curated list of protein complexes which have experimental evidence for physical binding, have been reconstituted in vitro, and which have some demonstrable molecular function [50, 51]. Compared to non-epigenes, epigenes are much more likely to be associated with at least one unique multiprotein complex (45% for epigenes; 13% for non-epigenes; *p* < 2.2e−16, 2-sample test of proportions) (Fig. 3B). Of genes that are known to associate in multiprotein complexes, epigenes tend to associate with a larger number of distinct complex species. For instance 65% of epigenes associated with a complex associate with more than one distinct complex compared to only 33% for non-epigenes, and 28% of epigenes participate in at least 4 unique complexes whereas the same is true for only about 5% of non-epigenes that exist in multiprotein complexes (Fig. 3C).

Related to the number of distinct complexes a gene's products associate with is the number of variable complex partners the gene's product has. Epigenes tend to have an intermediate number of distinct complex members in their associated complexes (geometric mean = 7.2, sd = 2.2) similar to that for non-epigenes associated with complexes (geometric mean = 6.3, sd = 2.9) (*p* = 0.79 permutation test) (Fig. 3D, Table S3). While roughly similar in terms of the number of distinct proteins that compose their associated complexes, epigenes have a significantly larger number of variable complex partners. Even when looking only at the 65% of epigenes and 33% of all non-epigenes which are associated with more than one complex, the number of variable partners is substantially greater for epigenes (geometric mean = 4.3, sd = 3.7) compared to non-epigenes (geometric mean = 1.7, sd = 2.4) (*p* < 1e−5, permutation test) (Fig. 3E) compared to all non-epigenes. Much of the broad range of epigene targeting and function can be explained by the additional layer of protein complexes with significantly increased numbers of variable binding partners.

### Extensive prevalence of disordered domains in epigenes

**Fig. 4:**
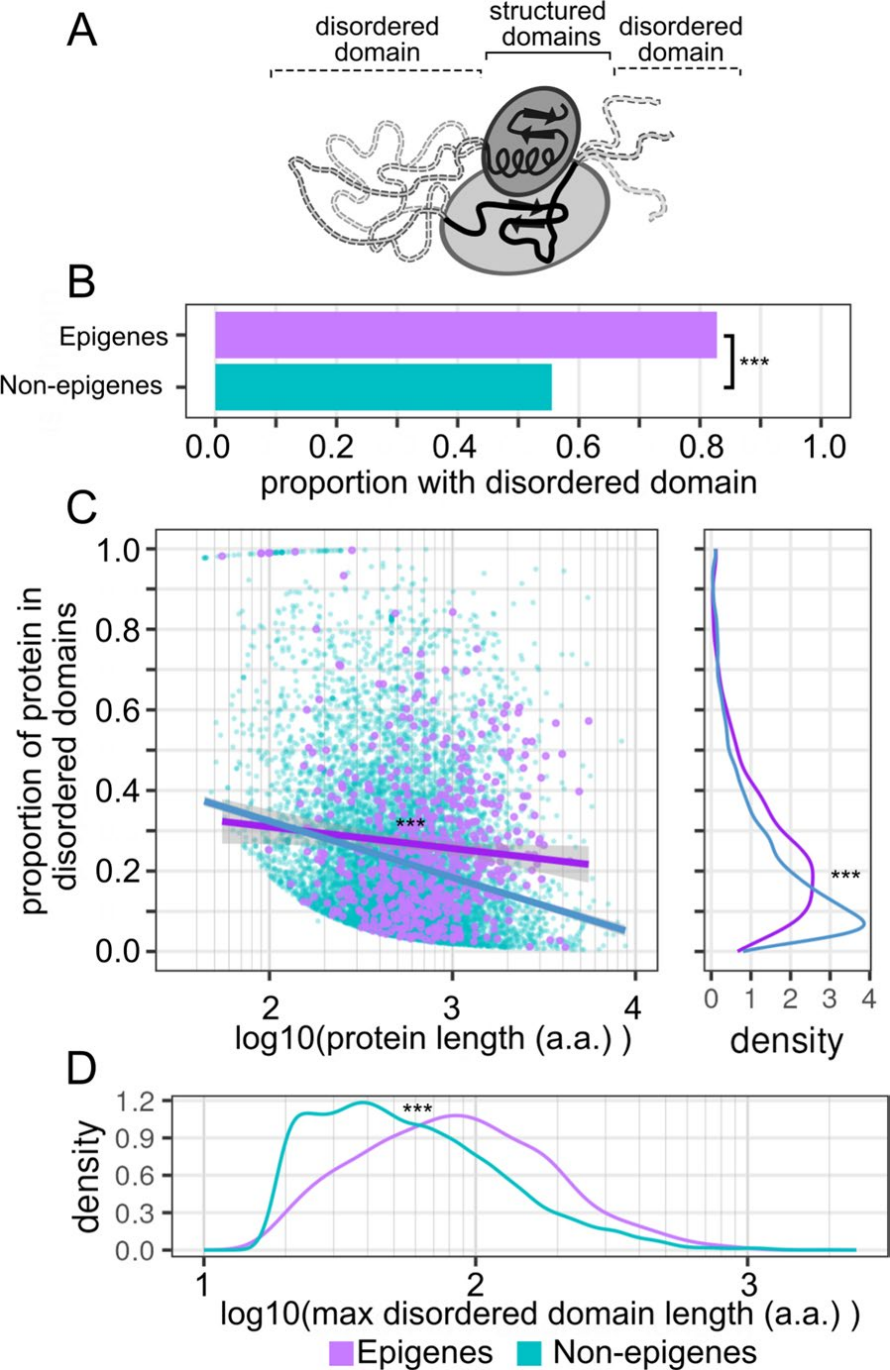
Epigenes are enriched in intrinsically disordered domains. **A** Toy diagram of disordered vs structured domains. While structured domains are relatively rigid, disordered domains are conformationally labile. **B** Proportion of genes with at least one disordered domain. **C** Proportion of protein that is annotated as belonging to a disordered domain. Each point is an individual gene which has at least one annotated disordered region. Regression lines shown for epigene and all other gene groups. Density of proportion of protein in disordered domain shown to the right of the scatterplot. Excludes all genes with no annotated disordered domains. **D** Density distribution of maximum disordered domain size for each protein with at least one annotated disordered region. Significance level is indicated by asterisks: NS = not significant, * < 0.05, ** < 0.01, *** < 0.001

Multiprotein complexes are just one of the types of functional aggregations that a gene's encoded products can contribute to, others, such as subcellular condensates and protein-RNA tethering, can also be critical to a gene's overall function. Recent studies have identified intrinsically disordered domains as critical to these roles enabling subnuclear organization of various epigenes [52] as well as interactions with RNAs [53, 54]. In contrast to structured regions of a protein, intrinsically disordered domains are characterized by a lack of rigid structure in their 3D organization (Fig. 4A). Based on the previously described potential for disordered domains to contribute to various facets of epigene function, such as in subnuclear organization or the targeting of specific genomic regions, we next tested whether disordered domains were common amongst epigenes.

To investigate disordered domains in epigene encoded proteins we pulled annotations from the UniProtKB resource [55, 56] in which disordered domain annotations are based on high confidence predictions from the MobiDB-Lite software [57]. In this dataset we observed that epigenes are more likely to contain a disordered domain than non-epigenes (83% epigenes; 55% non-epigenes; *p* < 2.2e−16, two-sample proportion test) (Fig. 4B). Additionally, of those genes which contain a disordered domain, a larger proportion of the epigene's encoded protein product falls in these domains (median = 22%, IQR = 13 to 36%) than for non-epigenes (median 16%, IQR 8 to 30%) (*p* < 1e−5, permutation test), and this proportion is more consistent between proteins of varying sizes whereas the expected proportion drops off more steeply for non-epigenes (Fig. 4C). Additionally, the largest contiguous disordered domain of epigenes tends to be substantially larger (median = 83 a.a., IQR = 47 a.a. to 144 a.a.) than that of non-epigenes (median = 51 a.a., IQR = 31 a.a. to 93 a.a.) (*p* < 1e−5, permutation test) (Fig. 4D, Table S4). It is widely known that nearly all histones contain a disordered tail domain. This domain, while shorter in absolute length than the disordered domain of most epigenes (median = 35 a.a., IQR = 22 a.a. to 45 a.a.) comprises a similar fraction of overall protein size (median = 28%, IQR = 17 to 31%) (Figure S3). These results are consistent with the overlap between the described functions of disordered domains and the processes which would intuitively seem important for epigenes to function, namely in enabling subnuclear organization of the protein complexes and in targeting protein complexes to specific regions of the genome via interactions with RNAs.

### Multivariate analysis of disordered domain content and protein complex structure

We next looked at some additional relations of disordered domain content with protein and protein complex size features (Figure S4). The regression of the overall average protein complex size, measured as the average number of distinct protein subunits per complex for the protein, to protein disordered domain content did not show a significant relation for either epigenes or non-epigenes. However, there was a modest positive correlation between the number of variable binding partners and the proportion of a protein in disordered domains for non-epigenes which was inverse for epigenes where a modest negative correlation was observed.

### Epigene associated disorders tend to follow dominant patterns of inheritance

Having looked at some of the sources of functional diversity in the species and structure of epigene encoded products, we next wanted to relate our results to the clinical outcome of perturbed epigene function or loss of function. Previous studies have shown an enrichment of epigene mutations in autism [58] and congenital heart defects [59, 60]. Our previous work showed that 20.6% of epigenes (148/720) cause at least one monogenic developmental germline syndrome [1] and the number of disease associated epigenes is substantially larger than previous reports with more stringent definitions for this class of monogenic disorders. They are often referred to as chromatinopathies [1, 61, 62] or epigenetic disorders of mendelian machinery [63], but the lists of included genes and syndromes are more restricted to epigenetic readers, writers, erasers and movers. Given their importance in monogenic disease, we next assessed the zygosity of genetic variants required for disease.

**Fig. 5:**
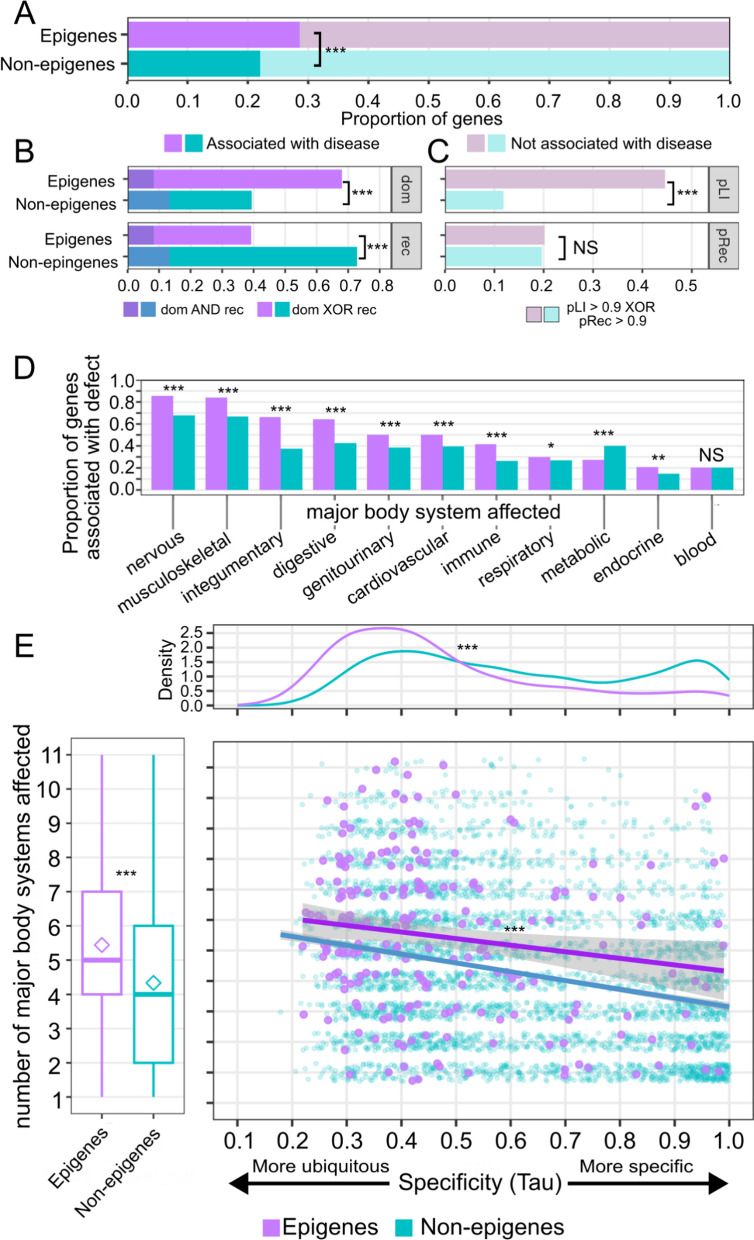
Epigene-associated monogenic disorders are characterized by dominant modes of inheritance and ubiquitous transcript expression profile across multiple body systems.** A** Proportion of genes associated with at least one mendelian disease. **B** Of genes associated with some mendelian disease, proportion associated with dominant and recessive modes of inheritance. **C** Of genes not associated with some mendelian disease, proportion associated with predicted dominant effects, pLI > 0.9, and predicted recessive effects, pRec > 0.9. **D** Of genes associated with some mendelian disease, proportion associated with some phenotype affecting the major body system indicated. **E** Center: Scatterplot of specificity of gene expression against number of body systems affected. Regression lines for epigenes and all other genes groups shown. *Left margin*: boxplot of number of major systems affected by each gene's associated diseases. Mean shown as diamond. *Top margin*: density distribution of specificity of gene expression. Significance level is indicated by asterisks: NS = not significant, * < 0.05, ** < 0.01, *** < 0.001

The Online Mendelian Inheritance in Man (OMIM) database reports associations with mendelian diseases for 29% (206/720) of epigenes compared with 21% of non-epigenes (4065/19,329). (*p* = 1.4e−6, 2-sample proportion test) (Fig. 5A, Table S5). Notably, a substantial majority of epigene associated mendelian disorders follow a dominant pattern of inheritance (epigenes 140/206 or 68%; all non-epigenes 1599/4065 or 39%; *p* = 6e−16, two-sample proportion test) (Fig. 5B). Consistent with this trend, for those genes not associated with any mendelian disease, epigenes are much more likely to have a high probability of loss of function intolerance (pLI), indicating probability of dominant-like deleterious effects upon loss of a single copy of the gene (pLI > 0.9: 229/514 or 45% for epigenes; 1809/15,264 or 12% for non-epigenes; *p* < 2.2e−16 two-sample proportion test) (Fig. 5C). Conversely, epigenes' associated disorders are less likely to follow a recessive inheritance pattern (epigenes 81/206 39%; non-epigenes 2959/4065 73%; *p* < 2.2e−16 two-sample proportion test) (Fig. 5B). However, of those genes not associated with any mendelian disorder, a roughly similar probability of recessive effects of gene loss (pRec) are predicted (epigenes 104/514 or 20%; non-epigenes 2996/15,264 or 20%; *p* = 0.78 two-sample proportion test) (Fig. 5C). Together these patterns of inheritance suggest that epigene function is dosage sensitive with a single gene copy being insufficient to maintain the normal development and health of the organism.

Histones genes in contrast remain largely unassociated with mendelian disease, with a few notable exceptions [64, 65]. Part of this may stem from the exceptional degree of genetic redundancy found in histones with each major histone having numerous genetic copies in the genome [38].

### Multiorgan phenotypes of epigene-associated syndromes

We next looked at the organ and body systems affected in epigene associated syndromes. When looking at the prevalence of phenotypes affecting each major body system, as defined by the human phenotype ontology (see “Methods” section). We found that epigene-associated syndromes (a.k.a. chromatinopathies, disorders of the epigenetic machinery) were more likely than non-epigenes to affect multiple body systems, with the exception of defects in blood/blood forming tissues, and metabolism (Fig. 5D, Table S5). The body systems most commonly affected in epigene syndromes were the nervous system and the musculoskeletal system, each found in over 80% of epigenes-associated syndromes. This is consistent with previous studies and highlights the role of epigenes in energy-intensive organs. Among all non-epigenes, 68% and 67% are associated with syndromes affecting the nervous and musculoskeletal systems respectively (*p* = 1.2e−7 and *p* = 8.2e−7 respectively, two-sample proportion test, bonferroni corrected for 11 tests) (Fig. 5D). Overall, epigene associated syndromes affect a greater number of body systems compared with non-epigene associated syndromes (*p* < 1e−5, permutation test) (Fig. 5E, left margin), which in part was reflective of the tendency of epigenes to be more ubiquitously expressed (*p* < 1e−5, permutation test) (Fig. 5E, top margin). However, even at similar levels of specificity of gene expression, the disorders associated with epigenes still had an inflated number of systems affected (Fig. 5E, center). Overall, the pervasive role of epigenes across multiple body systems cannot be entirely explained by their gene expression profile and likely is at least partially derived by their role in early developmental processes.

### Epigenes expressed in early neurodevelopment can be modulated by repurposed drugs

We next examined the gene expression patterns of epigenes in the developing human brain using bulk RNA-seq data from BrainSpan [66] as detailed in Figure S5. Only genes with expression quantified in the original BrainSpan analysis were included for our analysis (711 of the 720 epigenes, and 18,191 of 19,329 non-epigenes). From this analysis, we found that a majority of epigenes (693 of 711 mapped epigenes were expressed in at least one tissue and time point at 1 TPM or greater) were expressed in the human brain at some point between development through adulthood. Clustering epigenes based on their expression over prenatal brain development revealed that epigenes exhibit dynamic gene expression that increase, decrease, or oscillate overtime across brain regions (Figure S6A). We filtered the BrainSpan data to focus on gene expression occurring during prenatal development, which spanned post conception weeks 8–40 after filtering, to uncover how epigene expression varies along side genes critical for human fetal neurodevelopmental since pathogenic mutations in epigenes are enriched in pediatric syndromes characterized by intellectual disability and autism [67, 68]. Upon performing clustering analyses on the expression of epigene and non-epigene over prenatal brain development—we identified 97 metaclusters (MCs) composed of multiple gene clusters that display consistent co-expression across the developing human fetal brain (Figure S6A). Some of the most notable MCs were MC1, MC8, MC17, MC25, MC58, and MC92—which all decreased in expression across time as the genes in these MCs were significantly overrepresented in gene ontology (GO) terms related to cell cycle dynamics and proliferation (Figure S6B). Another notable set of MCs were those which increased gene expression over early brain development and were enriched for GO-terms relating to neurotransmission and synaptogenesis like MC13, MC18, MC23, MC61, and MC76 (Figure S6C). Importantly, both cell proliferation and synaptogenesis are core biological processes necessary for proper human brain development [69].

Finally, we wanted to identify perturbations that affect expression of epigenes, as a route towards rational drug discovery or repurposing. Moreover, it can assist with unraveling epigene function. For this, we looked at the L1000 dataset that quantifies the effects of 20,415 chemical perturbogens and drugs, on gene expression in 76 distinct cell lines and primary cell types [70] comparing these against matched cell types treated with DMSO vehicle as controls. We focused on the neural progenitor cell (NPC) here as we and others [71] have shown that epigene mutations are enriched for neurodevelopmental phenotypes, so we were most interested in identifying drugs which could modulate epigene expression in this context.

**Fig. 6:**
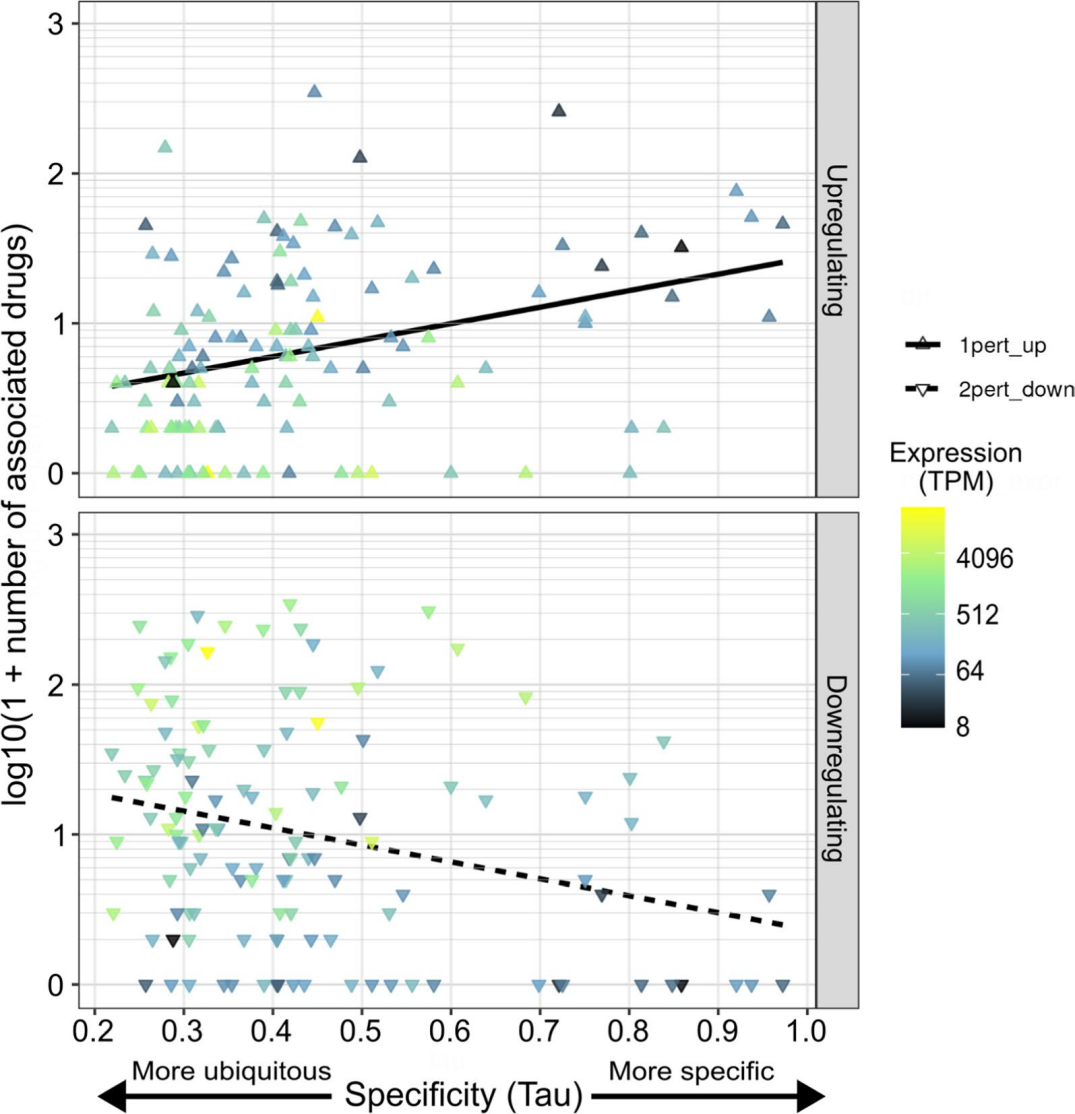
Drugs can substantially increase and decrease expression for specific epigenes associated with monogenic disorders, here shown for neural progenitor cells. Each point shown in both top and bottom panels is an individual epigene associated with some mendelian disease. Plotted on the x-axis is the gene's overall specificity of expression as measured by Tau, and on the y-axis for the top and bottom panels respectively are the number of drugs with at least a + 3 sd increase and a -3 sd decrease in the gene expression. Points are colored by the gene's overall expression level in the neural progenitor cells

In total 2620 drugs were associated with major expression level changes (± 3 sd from matched control expression distribution) of at least one epigene. All 591 epigenes that had their expression either directly measured or imputed in the L1000-NPC dataset had at least one drug associated with a change in their expression of at least 3 standard deviations from the epigene mean expression relative to the matched control distribution, the control distribution is described in detail in [70]. There were 489 epigenes with at least one drug associated with a > 3 sd increase in expression and 461 epigenes with at least one drug associated with a > 3 sd decrease in expression. The number of drugs associated with positive and negative change in expression for each epigene are shown against overall specificity of each epigene's specificity of expression are shown in Fig. 6. This figure also illustrates that, as intuition would suggest, a greater number of epigene-drugs interactions that push up expression are towards genes with otherwise low relative expression, and those that drive down expression are towards genes with otherwise at least moderate relative expression. A table of all large effect epigene-drug interactions is provided in Table S6, as well as a summary table of the number of genes (both epigenes and non-epigenes) affected by each drug affecting at least one epigene (Table S7), and a summary table of number of drugs increasing and decreasing expression of each measured or imputed epigene by at least 3 sd (Table S8).

While this dataset provides insight into drugs that can modulate epigene expression, deeper investigation will be required to dissect out whether the drugs act in a direct or indirect manner, the associated specificity of the drug for one or more genes or pathways, and the actions across multiple cell types or tissues. Given the structural diversity of epigenes and their complexes, dissecting out the effects of these drugs will enable improved targeting and chemical modifications to target the isoform and/or complex of interest.

Taken together with the in vitro L1000 NPC data, the dynamic in vivo temporal expression of epigenes in the human brain provides valuable computational resources that could offer therapeutic avenues for neurodevelopmental disorders caused by epigene mutations, a concept supported by recent reviews in the field highlighting pre- and post-natal neural malleability [63]. Moreover, given the widespread use of small-molecules targeted at epigenes, understanding the impact across the organ systems will allow for improved assessment of off-target effects and potential side-effects in these patients.

## Discussion

Our work is a comprehensive assessment of how epigenes enable a huge diversity of functions across multiple organ systems. Our study shows that epigenes are larger in gene and transcript size, and this increased size is due to increased number of exons. The functional diversity comes from two sources: more annotated isoforms and from the possible combinations between complex members that control context specificity of their epigenetic reader, writer, and eraser functions. Moreover, we found that intrinsically disordered domains are both larger as contiguous regions and make up a greater proportion of the individual epigenetic protein products for epigenes, and epigenes have a greater number of variable multiprotein complex binding partners relative to non-epigenes. Together, these findings highlight how a small number of genes can coordinate a diversity of cell-types, tissues and responses to environmental stressors.

This analysis is based on publicly available transcriptomic, genomic, and proteomic databases that are curated based on what has been known and published. Therefore, it is limited in its ability to assess direct and mechanistic effects of epigene mutations. What we have learned is the complexity of its function is derived from structural, isoform, and complex partners that drive their vast functions. However, we recognize that there are likely additional genes whose epigene functions remain undiscovered and were classified as “non-epigenes”. There are many different definitions one can use to classify genes that control epigenome function: chromatinopathy [1], Mendelian disorders of epigenetic machinery [9], and others and we based our choice on previously published work that encompassed a broader definition of epigenes [28] with the goal of ensuring we captured those with supporting non-enzymatic functions in addition to the more classically defined chromatin modifiers distinguished by their having enzymatic functions.

We began by looking at functional diversity of epigenes and their gene structure and relating this to their isoform expression variety. Alternative splicing is a widely recognized mechanism by which genes achieve increased functional diversity [22]. Differential isoform usage contributes to distinct cellular and tissue identities, for instance by including protein domains critical to function in one context but not another [22, 72], modifying transcript and protein localization [73, 74], or by altering the kinetics of translation of RNA to protein modulating the gene dosage with respect to the biological context in which it is expressed [72, 75, 76]. We were intrigued to find that epigenes were, at the simplest level, larger at the genomic and transcriptomic levels and had an increased number of exons and annotated transcripts. One concern is the possibility that the increased number of isoform annotations might be coming from very lowly expressed transcripts that are not functional but rather transcriptional noise or sequencing errors. To get around this we incorporated weights to our measures of intratissue entropy and intertissue divergence which gave more weight to those tissues where the gene was more highly expressed. In addition to distinct isoforms of a given gene, a gene may also have paralogs with more or less overlapping functional niches, reflecting functional degeneracy, a concept reviewed for epigenetic systems in detail in [77]. In our regression analyses we observed a slight but significant decrease in intratissue entropy together with a slight but significant increase in intertissue divergence for both epigenes and non-epigenes. As intratissue entropy is an effective measure of the number of expressed isoforms within a tissue, and intertissue entropy is a measure of how different the isoform patterns are between tissues, these results are consistent with the hypothesis that isoforms and paralogs can act as alternative paths towards functional degeneracy. The correlation of paralog count to overall gene expression specificity, while positive for both epigenes and non-epigenes, was significantly less strong for epigenes than non-epigenes. One explanation for this might be related to the functional diversity of epigenes existing with respect to genomic diversity as opposed to tissue or cell type diversity. Future studies including deeply sequenced long-read transcriptomic data should be assessed to validate the isoform level differences.

Beyond the functional diversity from differential isoform usage, we showed that epigenes have a far larger number of variable binding partners than non-epigenes. The function of multiprotein complexes is defined by their constituent proteins, so by binding a larger number of variable partners and participating in a greater number of compositionally distinct complexes epigenes can extend the range of their functionality [25, 26]. The effects that these different complexes have are poorly understood, but in general they may target distinct genomic regions, produce different epigenetic modifications, or act in response to different cellular signals. Future work will be necessary to unravel the different functions of individual complexes, however investigating this functional diversity will not be trivial even with existing methodologies. Many of these epigene complexes are known to share binding partners with one another, and so isolating the effects of individual proteins and complexes is a major challenge. For instance, KAT6A's three unique complexes, which are characterized by the BRPF homolog they incorporate, largely mirror the complexes of its own homolog, KAT6B [27]. Thus, experiments aimed at distinguishing the role of KAT6A-BRPF1 against KAT6A-BRPF2 or KAT6A-BRPF3 based on, for instance, knocking down BRPF1 will also affect the KAT6B-BRPF1 complex. One potential solution to investigate the function of specific complexes, such as KAT6A-BRPF1, might be to generate artificial fusion products that are analogous to these endogenous complexes. Designing and validating such fusions is far from trivial, however with the rapid advancement of protein structure prediction tools [78] it may soon be feasible to design high-throughput experiments that can generate such fusion products and then investigate their isolated functions.

Beyond protein–protein interactions and the formation of multiprotein complexes, the domains on epigenes provide an additional handle by which their function is modulated. We found that epigenes encode proteins that are more likely to include disordered domains, and that these domains of disorder tend to be substantially longer than those of other proteins. Our findings align well with previous work [52] that identified disordered domains as critical for functions that are intuitively important for epigene function such as subnuclear localization and the formation of subnuclear regulatory structures and, relatedly, interactions between proteins and diverse but specific subsets of long non-coding RNAs (lncRNAs). Intrinsically disordered domains are cited as being enriched among hub proteins, those proteins with many interaction partners [79, 80]. This seems to be in conflict with our multivariate regression analysis where we observed that epigenes have a negative correlation between proportion of disorder and the number of variable binding partners while non-epigenes have a substantial positive correlation. This may apparent contradiction with previous results may be due to a number of factors, for instance an artifact of epigene disordered domains having weaker binding with other proteins which more often goes undetected, or perhaps epigene disordered domains more often being employed for protein-RNA as has been reported for instance for some polycomb repressive complexes [81], as the EMBL-EBI Complex Portal we employed for annotations does not include protein-RNA interactions.

Similar to our findings in our isoform analysis, Fig. 2, we showed that epigenes tend to be more ubiquitously expressed than non-epigenes. While this ubiquitous expression helps to explain the tendency for epigene related syndromes to affect diverse body systems, we also found that epigenes had greater phenotypic complexity than non-epigenes even after accounting for the specificity of their expression. Epigenes are critical in the regulation of genetic information and so changes in their functions will likely propagate to additional genes and their own consequent pathways [82, 83]. Epigene's function as hubs of regulation therefore can explain why their associated disorders have greater levels of complexity even than non-epigenes with similar levels of expression specificity.

Finally we looked at some of the tools that exist for modulating epigene expression. While we highlight a variety of drugs identified in the L1000 dataset [70] that affect epigene transcription levels, more or less specifically, further work will be necessary to characterize the specificity of these drugs for distinct isoforms and species of multiprotein complexes in which epigenes function. Other tools such as CRISPR provide another means of controlling which epigenes are present to test for individual effects, but further advances will be necessary to parse out the functions of the diverse species of epigenes and their variants that exist in the organism.

## Methods

### Definition of gene sets

Epigenes were defined as those belonging to the epigene list in [1] (n = 720). The contrast set included in this study was restricted to all protein coding genes that were not epigenes, called the non-epigenes throughout, obtained from Ensembl BioMart [39, 84] (version GRCh38.14) (n = 22,494). Histones gene annotations (n = 100) were obtained from Ensembl BioMart (version GRCh38.14).

### Annotation of gene structure

Structural annotations for genes, transcripts, and exons were obtained from Ensembl BioMart [39, 84] (version GRCh38.14) using a filter to only include protein coding genes with at least one annotated transcript (n = 19,329). For the analysis of gene structure and exon length, only the canonical isoform annotation was used for each gene, i.e. *transcript_is_canonical* attribute was set to *TRUE*. For the isoform analyses, all Ensembl annotated isoforms were used.

### Permutation tests

Permutation tests between the epigene and non-epigene sets were performed as follows. The non-epigene set was randomly sampled without replacement to generate a contrast set equal in size to the epigene set for each respective test. This was repeated up to 100,000 times for each test, setting a lower bound of detectable significance at 1e−5 (i.e. 1 in 100,000). For two-sided tests a simplifying assumption of symmetry was made in estimating significance of a difference between groups. For instance if 1 in 20 cases the test statistic of group1 was higher than group2 (19 in 20 for the converse), the more extreme observed 0.05 would simply be double to obtain *p* = 0.10.

### Quantification of entropy and divergence

Isoform expression estimates were obtained from GTEx V8 [43, 44]. Transcript abundance predictions available through GTEx are based on bulk short-read RNA-seq data and are determined by the RSEM algorithm [45].

For the analysis of isoform usage two distinct metrics were used. The first is for within-tissue or within-biosample diversity of isoform usage, a measure for distinguishing between genes with many isoforms expressed in a given context and those with few. The other is for between-tissue divergence of isoform usage, to distinguish between genes that have more distinct isoform usage patterns between tissues or biosamples and those with more similar isoform profiles.

Both the intra-tissue entropy and inter-tissue divergence are weighted measures using, for a given gene, a common pair of weight vectors. The first weight used incorporated tissue-tissue similarity to make estimates more robust to oversampling of highly similar tissues as described in [85]. The equation used to calculate this weight is given as:1

Here *w*_*t*,1_ is the sample set similarity weight for tissue *t*. *d*_*l*,*p*(*t*)_ is the distance between node *t* and its parent node *p*(*t*). *v*_*t*_ is the number of leaf nodes descendant from node *t*. *w*_*p*(*t*)_ is then the recursively determined weight of the parent node. Prior to calculating the weights, tissues or biosamples must be hierarchically clustered. For this we used the same method described in [85]. This weighting scheme is analogous to one we previously used for balancing estimates of transcriptomic specificity [85] which itself was developed from the weighting method implemented by the CLUSTALW multiple sequence alignment CLUSTALW algorithm developed by [86].

The second weight incorporates expression information to reduce the weight of biosamples that have low relative levels of expression for the given gene. This weight is calculated as:2

Here *w*_*t*,2_ is the relative expression weight for tissue *t*. *ε*_*t*,*i*_ is the median normalized log10 expression value for isoform *i* and *n* is the total number of unique isoforms for the given gene that are expressed in at least one tissue. *m* is the total number of unique tissues or biosamples in the overall sample set. Thus *w*_*t*,2_ is simply the proportion of overall expression in the given tissue *i* relative to maximum expression across all tissues or biosamples.

The product of these two weights incorporating both sample similarity information and relative expression information gives the final weight attributed to the biosample for downstream calculation in both entropy and divergence measures. This is given as:3

To measure within-tissue diversity of isoform expression, we used the weighted mean entropy of isoform proportions within each tissue. Entropy balances the number of expressed isoforms with their relative level of expression, such that minimum entropy is achieved when a single isoform dominates and maximum entropy is achieved when all expressed isoforms have equal expression.

The basic implementation of entropy used is provided as:4where *N*_*t*_ is the indices for the set of isoforms with non-zero expression in tissue *t*, and *x*_*t*,*i*_ is the proportion of expression for isoform *i* out of all isoforms expressed in tissue *t*. To clarify this notation we note that:5

Using the definition of entropy in Eq. (4) and the tissue or biosample weights defined in Eq. (3), we then calculate the weighted mean entropy across all *m* tissues as:6

To measure between tissue diversity of isoform profiles, we use the Kulback-Leibler divergence (*D*_*KL*_) [49], to measure the mean divergence of isoform expression profiles from the typical, that is mean, isoform expression profile. The mean isoform expression profile is defined as:7where:8

Here *q*_*i*_ is the weighted mean proportion of isoform *i*, with *w*_*t*_ and *x*_*t*,*i*_ defined as above. The *D*_*KL*_ for each tissue *t* is then calculated as:9

and the weighted mean of this is provided as:10

### Multiprotein complex analysis

The list of multiprotein complex associations was obtained from the EMBL-EBI Complex Portal which is restricted to high confidence protein complexes with experimental validation of physical interactions, demonstrated in vitro complex reconstitution, and some verified molecular function [50, 51].

### Analysis of disordered domains

Annotations for protein disordered domains were obtained from UniProtKB [55, 56] (2024_04 release). UniProtKB annotations for disordered domains come from based on high confidence predictions from the MobiDB-Lite method [57].

### Syndrome phenotype analysis

Gene-syndrome-phenotype annotations were obtained from the online mendelian inheritance in man (OMIM) resource [87, 88]. Phenotypes associated with individual genetic disease in OMIM are annotated with human phenotype ontology (HPO) [87, 89] terms which enables mapping higher resolution concepts, such as atrial septal defect (HP:0001631) to their more general parent terms such as cardiovascular system defect (HP:0001626). Using the structure of the HPO we were therefore able to map each individual disorder to the set of major body systems they affected. Major body systems were defined as a subset of the HPO terms that are direct children of the HPO term for Phenotypic abnormality (HP:0000118).

Additionally, here we limited our analysis to germline mendelian diseases, filtering out any diseases that were annotated in the OMIM as resulting from somatic mutations which mostly represented associations with cancers. Genes associated with phenotypes as susceptibility factors were also not included in this analysis.

### Expression data acquisition and processing

RNA-seq expression data was compiled from both the Gene-Tissue Expression (GTEx) project and from non-overlapping datasets available through the ENCODE Consortium filtered for human primary tissues and cells.

The GTEx data used was the median expression data matrix, and data from ENCODE was also aggregated by taking the median of expression across samples of a common biosample type, i.e. cell or tissue of origin. As our analyses of gene specificity were performed at fairly coarse resolution, we used the simplifying assumption that for most genes, the biosample type would be the primary source of variance in gene expression, and factors such as age and sex would be minor. As [90] found that intertissue variance is far greater than factors such as age and sex, we believe this simplification is justified.

Normalization of gene expression was performed as follows. First, non protein coding genes and mitochondrial genes were filtered out of the expression matrices. All expression values were then renormalized to TPM using the protein coding gene set.

Following conversion to TPM, as the distribution of gene expression is approximately log normal, the expression values were log10 + 1 transformed. Finally, we performed median normalization on the log10 transformed TPM values, which enables a more robust comparison between distinct biological samples that may have dramatic differences in expression level of a few genes that shift the base TPM distribution for all others [91].

To calculate specificity of gene expression we used a weighted measure of Tau which we describe in our previous work [85]. This weighted measure was developed from the Tau statistic, initially proposed in [92], to incorporate sample similarity information to avoid over-represented biological samples from skewing estimates of expression specificity.

### Brain span analysis

Bulk RNA-seq data from BrainSpan [66], spanning embryonic week 8 to week 40 (w8 to w40) and postnatal development (p0-y40) across 26 distinct brain tissue subregions and 52,736 genes, was processed and normalized as follows. Samples were first grouped by subregion and ordered by ascending post-conception weeks (pcw) up to w40. Expression values from samples of the same age within a subregion were averaged, converted from RPKM to TPM.

The protein-coding gene set was aligned to the BrainSpan dataset to define the overall gene universe, leading to the identification of 711 epigenes with mapped Ensembl IDs. Clustering analysis was conducted for brain subregions with at least five time points sampled (17 out of 26 subregions). Genes with a temporal expression range below 0.5 were excluded due to minimal expression variation over time. Maximum expression for each gene was scaled across all time points to facilitate comparison of expression patterns across genes. Epigenes were clustered using average clustering based on embryonic expression profiles. A weighted Pearson's correlation was then employed to cluster epigenes with similar expression patterns. The weights used were based on log scale time bins with each bin having equal weight distributed across all samples within the bin. For our analysis, 4 bins spanning 10 weeks of development each from conception to birth (week 0 to week 40) were used to account for uneven sampling across developmental timepoints. Subsequently, non-epigenes were assigned to the cluster that best matched their expression profile (time correlation > 0.8).

Following the clustering for each brain subregion, tissue clusters were further clustered based on similar mean expression trajectories over time. Missing timepoint expression values were imputed to accurately compare clusters across tissues using the expression value from the nearest time point. The resulting “metaclusters” were characterized by average temporal expression and Gene Ontology (GO) enrichment analysis, with gene sets identified based on a dynamic tissue membership threshold (Figure S5).

## Supplementary Information

Additional file 1.

Additional file 2.

Additional file 3.

Additional file 4.

Additional file 5.

Additional file 6.

Additional file 7.

Additional file 8.

Additional file 9.

## Data Availability

All code for analysis performed in this work is available on GitHub at: https://github.com/leroybondhus/chrom_mod_expr_landscape.

## Code availability

All code for analysis performed in this work is available on GitHub at: https://github.com/leroybondhus/chrom_mod_expr_landscape
