# Supplementary material for: Epigene functional diversity: isoform usage, disordered domain content, and variable binding partners
Source: Epigenetics Chromatin. 2025 Feb 1;18:8. doi: 10.1186/s13072-025-00571-z (PMC11786378; doi:10.1186/s13072-025-00571-z)
Supplement: Supplementary file 1 — Additional file 1. [file 13072_2025_571_MOESM1_ESM.docx]

In preparation for

Epigenetics

Epigenetics and Chromatin

**SUPPLEMENTAL FIGURES FOR: Epigene Functional Complexity: Isoform Diversity, Disordered Domain Content, and Variable Binding Partners**

Leroy Bondhus^1,2,3^, Aileen A. Nava^1,2,3^, Isabelle S. Liu^1,2,3^, Valerie A. Arboleda^1,2,3,4,5*^

1 Department of Human Genetics, David Geffen School of Medicine, UCLA, Los Angeles, CA 90095

2 Department of Pathology and Laboratory Medicine, David Geffen School of Medicine, UCLA, Los Angeles, CA 90095

3 Department of Computational Medicine, David Geffen School of Medicine, UCLA, Los Angeles, CA 90095

4 Molecular Biology Institute, UCLA, Los Angeles, CA 90095

5 Jonsson Comprehensive Cancer Center, UCLA, Los Angeles, CA, 90095, USA, Los Angeles, CA 90095

*Corresponding Author

Valerie A. Arboleda, MD PhD

615 Charles E. Young Drive South

Los Angeles, CA 90095

310-983-3568

[varboleda@mednet.ucla.edu](mailto:varboleda@mednet.ucla.edu)

**Keywords:** transcriptomics, epigenes, chromatin modifiers, epigenetics, rare diseases


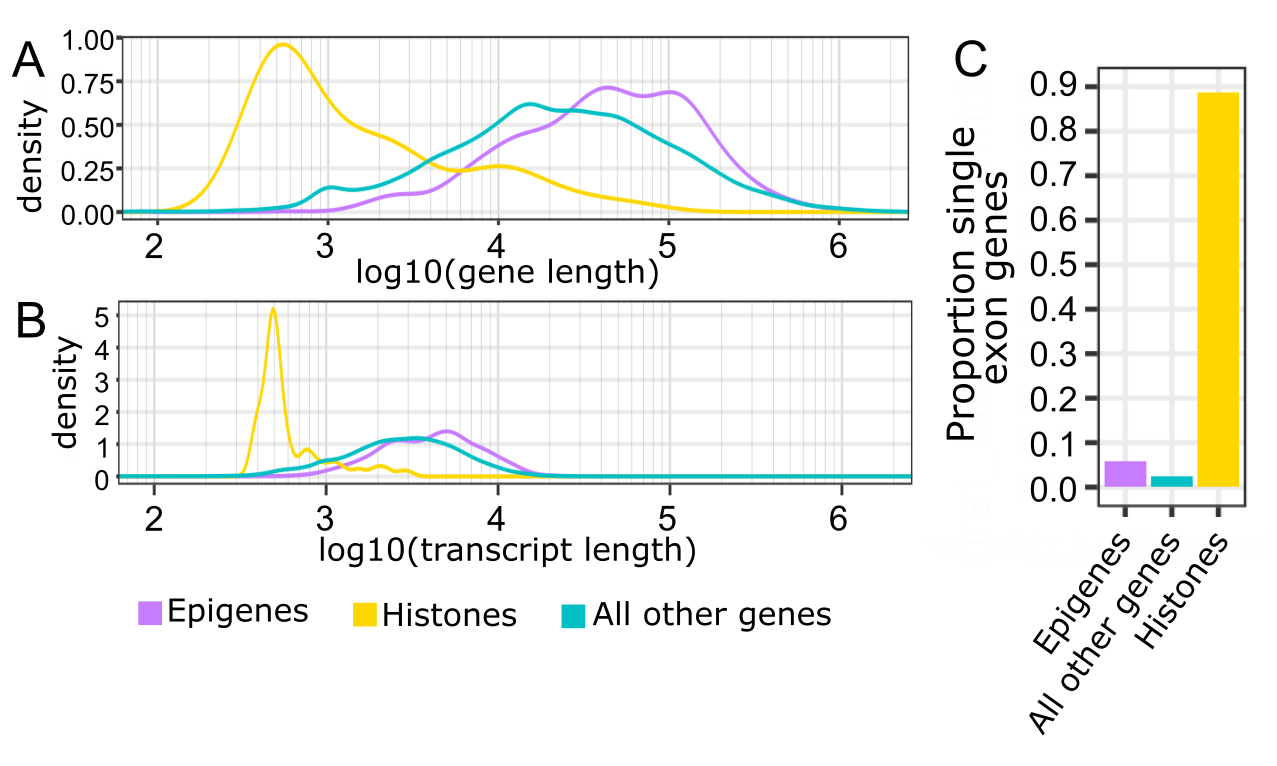


**Supplemental Figure 1. Comparison of epigene and histone gene and transcript architecture.** **A)** Density distribution of gene lengths for histones, epigenes, and all other genes. **B)** Density distribution of transcript lengths for histones, epigenes, and all other genes. **C)** Proportion of histones, epigenes, and other genes that are encoded by single exon genes.


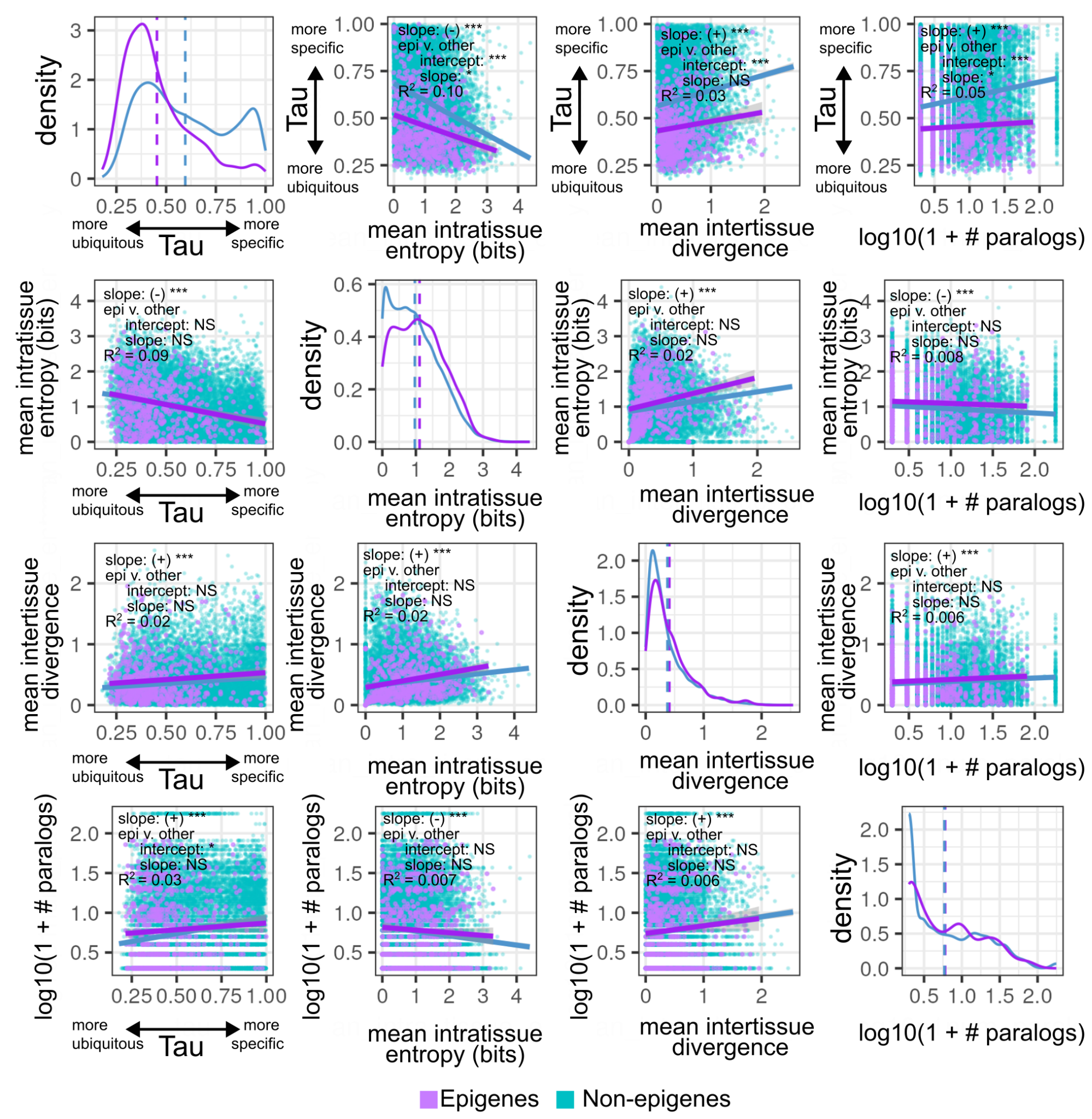


**Supplemental Figure 2. Multivariate analysis of gene expression specificity (Tau), intratissue entropy, intertissue divergence, and number of gene paralogs.** Summary of the simple linear regression, *lm( y ~ x + is_epigene + x:is_epigene)*, is reported within each scatterplot comparing each variable pair. Significance level is indicated by asterisks: NS = not significant, * < 0.05, ** < 0.01, *** < 0.001, bonferroni-corrected for 12 tests. Tau is a measure of gene expression specificity, described in detail in the main text and methods. Mean intratissue entropy is a measure of isoform expression diversity within a given tissue or cell type, described in detail in the main text and methods. Intertissue divergence is a measure of isoform expression diversity between different tissues or cell types, described in detail in the main text and methods. The number of paralogs associated was obtained from Ensembl BioMart (version GRCh38.14) with 25% sequence identity used as a cutoff to call paralogs. If either of two genes had 25% sequence identity between each other, the pair were both called paralogs. Regression lines shown for each pair of covariates. For consistency with the main text, only genes with more than one annotated isoform are included in this analysis.


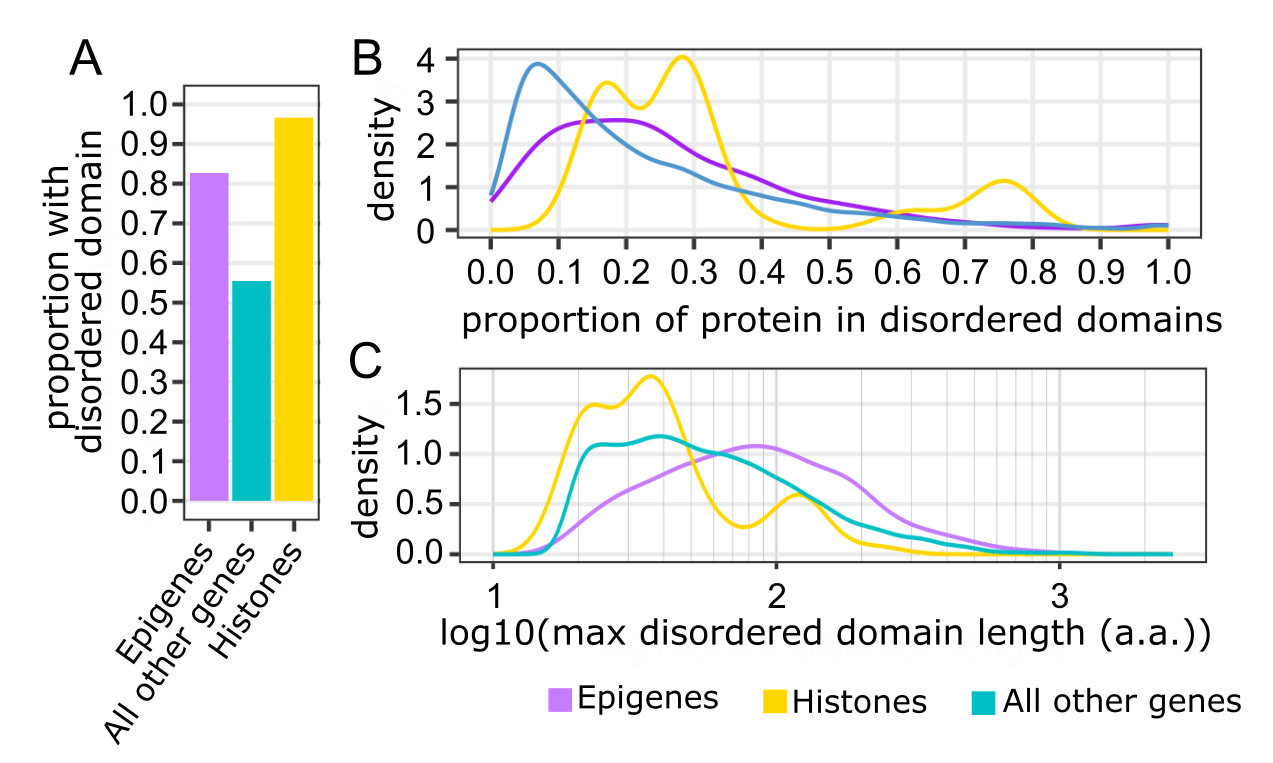


**Supplemental Figure 3. Comparison of epigene and histone protein disordered domain characteristics.**  **A)** Proportion of genes with at least one disordered domain. **B)** Density distribution of proportion of protein in disordered domains. Excludes all genes with no annotated disordered domains. **C)**  Density distribution of maximum disordered domain size for each protein with at least one annotated disordered region. Excludes all genes with no annotated disordered domains.

**
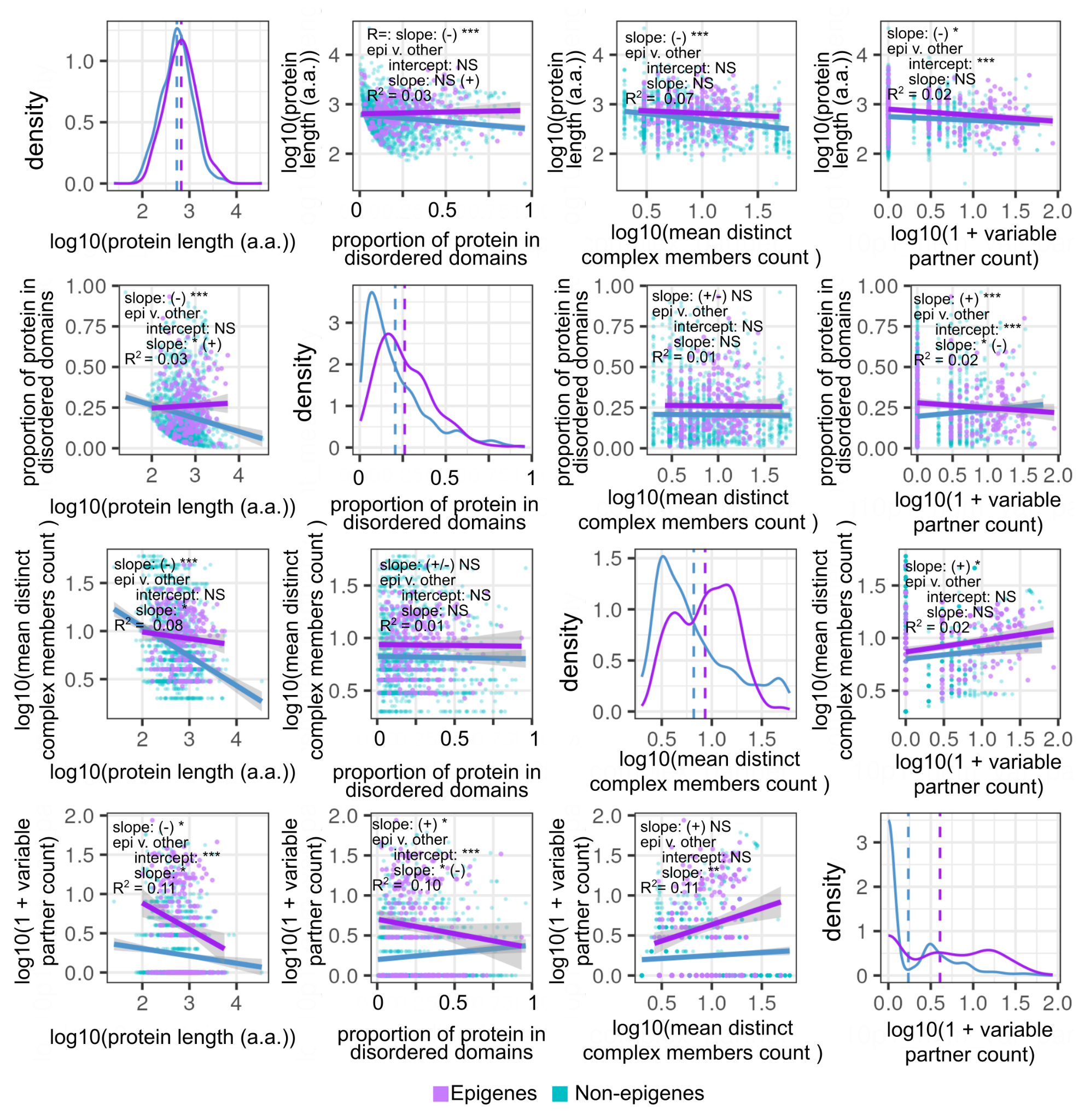
**

**Supplemental Figure 4: Multivariate analysis of protein complex characteristics and protein disordered domains.** Summary of the simple linear regression, *lm( y ~ x + is_epigene + x:is_epigene)*, is reported within each scatterplot comparing each variable pair. Significance level is indicated by asterisks: NS = not significant, * < 0.05, ** < 0.01, *** < 0.001, bonferroni-corrected for 12 tests. Covariates are the same as those described in Figures 3 and 4 of the main text. *Mean distinct complex members count* is the average number of unique proteins a gene's protein product is annotated as co-occuring in a functional complex with. *Variable partner count* is the number of unique proteins a gene's protein product is annotated as co-occurring with in some but not all of its member protein complexes. See Figure 3A for graphical summary of how counting was done.



**Supplemental Figure 5. Workflow for analyzing epigene and non-epigene expression within human fetal brain tissue from BrainSpan bulk RNA-seq database.** For high-resolution clustering, a threshold cutoff height of 0.2 was selected, resulting in the identification of 97 metaclusters; note since each W cluster may come from a different brain region, resulting metaclusters vary in size.



**Supplemental Figure 6.** **Association of epigenes with temporal dynamics of gene expression during human fetal brain development spanning 0 to 40 post conception weeks (pcw).** **(A)** From our metaclustering analysis, we identified 97 metaclusters in which epigene expression was similar to other non-epigenes in the developing human fetal brain. Here we show the average gene expression pattern for 30 of 97 metaclusters identified in our analyses to illustrate distinctive characteristics of these gene modules. Notably, since several large metaclusters followed similar expression patterns across developmental time, we performed Gene Ontology (GO) enrichment analyses on the genes which composed each metacluster -- finding these metaclusters tended to be enriched for **(B)** cell cycle/proliferation-related genes and **(C)** neurodevelopment/synaptogenesis-related genes.
